# Homestay businesses’ strategies for adapting to and recovering from the COVID-19 pandemic: A study in Vietnam

**DOI:** 10.1177/14673584221103185

**Published:** 2022-05-19

**Authors:** Tin Doan, Richard Aquino, Hongxia Qi

**Affiliations:** Business School, 4918 University of Greenwich, UK; 2496 University of Canterbury, New Zealand; 8491 Victoria University of Wellington, New Zealand

**Keywords:** adaptation strategy, business resilience, crisis recovery, homestays, service interaction, COVID-19, Vietnam

## Abstract

Adaptation and recovery are essential for businesses to survive crises and disasters. Drawing on the concepts of business resilience and hospitality service delivery practices, this study explored strategies employed by owners of Vietnamese homestay businesses for adapting to and recovering from the COVID-19 pandemic. Semi-structured interviews with 17 purposefully selected homestay owners were conducted during the pandemic. The thematic analysis of the data revealed three major strategies: adapting operational protocols, modifying products and service offerings, and reinstating the authenticity of the homestay experience. A conceptual model illustrating the transformations in homestay businesses was developed from the findings. As the study particularly revealed that the selected Vietnamese homestay businesses showed a strong capacity for adaptation, we discuss several factors influencing the implementation of adaptation and recovery strategies. This study contributes to the understanding of how micro, small, and medium-sized enterprises can adapt to external shocks and new externalities, and provides practical implications for homestay owners and stakeholders in relation to their post-pandemic business recovery.

## Introduction

The global tourism and hospitality industry is subject to the negative effects of disasters and crises such as the COVID-19 pandemic. The characteristics of the tourism industry make it particularly vulnerable to the effects of external shocks, particularly in the micro, small, and medium-sized enterprises (MSMEs) that comprise multiple sectors of the industry (Biggs et al., 2012; Ha et al., 2020). The ability of these businesses to adapt to surprises is identified as particularly important in such turbulent situations and prolonged crises (Ritchie, 2009; Williams et al., 2017) as presented by the pandemic. Harnessing business resilience by building the capacity to adapt in a rapidly changing environment is critical for the success and continuity of tourism businesses (Hinson and Slade, 2011).

A growing body of literature has developed on understanding business resilience (e.g., Sarker et al., 2020), especially during the COVID-19 pandemic. Resilience can be understood as short-term coping responses and long-term adaptation strategies (Speelman et al., 2014), as well as proactive and reactive strategies (Mayson and Barrett, 2017) implemented by organizations and individuals. Research on resilience in the tourism context has largely focused on destinations’ adaptation to climate change (e.g., Kaján and Saarinen, 2013; Njoroge, 2015), and tourism and hospitality businesses’ resilience, particularly their response, adaptation, and coping strategies to crises and disasters (e.g., Dahles and Susilowati, 2015; Prayag, 2018; Sharma et al., 2021). Since most tourism and hospitality businesses are considered MSMEs, such enterprises are also the organizations most affected by the negative impacts of disasters and prolonged crises (e.g., Thukral, 2021). Exploring how tourism and hospitality MSMEs adapt to these impacts and how can they build long-term business resilience is therefore an important undertaking. Taking the case of homestay businesses, this study focuses on how tourism and hospitality MSMEs transform their processes in order to adapt to the changes and disruptions brought about by crises.

Homestays are popular tourism and hospitality enterprises providing accommodation services in many rural destinations. In Vietnam alone, it is estimated that the numbers of homestay providers in Ho Chi Minh City and Ha Noi sharply increased from about 8,000 to more than 30,000 homestays between 2017 and 2019 (Uyen, 2020). As a type of home-sharing business, homestays are usually family-owned and operated businesses offering hospitality services such as rooms, food, and beverage, and authentic cultural experiences with local hosts (Ibrahim and Razzaq, 2010; Mura, 2015). These businesses are regarded as community-based enterprises, promoting sustainable community development through the involvement of local residents as tourism entrepreneurs (Pasanchay and Schott, 2021). By providing tourism services in their private dwellings, homestay owners can improve their livelihoods, as these businesses become their main or supplementary income sources (Truong et al., 2014). As MSMEs, their typical characteristics, such as limited personnel and lack of crisis management planning, render them vulnerable to the negative impacts of external shocks, and limit their ability to respond to crises (e.g., Pham et al., 2021). Therefore, it is vital to determine how homestay businesses adapt to external environmental changes, and how these enterprises might recover from the impacts of the prolonged COVID-19 pandemic.

Research on the resilience of tourism and hospitality organizations against crises such as the COVID-19 pandemic has been emerging since the pandemic started (e.g., Sharma et al., 2021). In the accommodation sector, recent studies have investigated the responses and adaptive mechanisms of large hotels, particularly in terms of their human resource practices (Su et al., 2021), general managers’ responses (Giousmpasoglou et al., 2021), corporate social responsibility (Marco-Lajara et al., 2021), and marketing and promotions, (Hoang et al., 2021), However, although research has been conducted on the resilience of rural hotels during COVID-19 (e.g., Marco-Lajara et al., 2021), there are no scholarly investigations into the adaptation strategies of homestay businesses and their service delivery processes, leaving a significant knowledge gap. Drawing on the concepts of business resilience and hospitality service delivery practices, this study aims to explore the strategies for adapting to and recovering from the COVID-19 pandemic. By analyzing data collected from Vietnamese homestay owners, the study addresses the following research questions:

• How do homestay businesses adapt their processes to the effects of the COVID-19 pandemic?
• What strategies could homestay business owners implement for post-pandemic recovery and to build long-term resilience?

The study employed a constructivist qualitative research methodology to make sense of narrative data collected from Vietnamese homestay owners during the COVID-19 pandemic. The findings add to existing conceptualizations on building the resilience of tourism and hospitality MSMEs, and addresses the call to re-imagine tourism home-sharing businesses facing new externalities such as COVID-19 (e.g., Lim et al., 2021). The exploration of this issue is significant from a theoretical standpoint, but also, and most importantly, from a practical standpoint. Insights from this inquiry will be valuable to homestay owners and their stakeholders, especially with terms of building their adaptive capacities during and after prolonged crises.

## Literature review

### Homestay businesses: Structure, product offerings, and service delivery practices

Homestays are accommodation enterprises in which private home owners utilize their spare space for business purposes. In other words, homestay operators commercialize residential homes for profit (Kontogeorgopoulos et al., 2015). This definition of a homestay is aligned with the concept of the commercial home enterprise proposed by McIntosh et al. (2010), while sharing many of the characteristics of conventional accommodation services.

Although the literature discusses various types of homestays, they are generally considered a type of home-sharing business (e.g., Lim et al., 2021). Homestays are tourism and hospitality MSMEs providing multiple services, including accommodation, food and beverage, and recreational activities (Truong et al., 2014). Homestay hosts can offer experiences that enable them to stand out from other accommodation providers by integrating the cultural elements of their homes with products and services (Ngo and Doan, 2014). Guests who share spaces with their host families can enjoy access to private dwellings and the learning that arises from being immersed in the local lifestyle (Truong et. Al., 2014). This is considered as a key component and benefit of the homestay guest experience. Instead of experiencing the standardized services of mainstream accommodation, interactions with homestay service providers can offer guests the opportunities to meet and learn from the lifestyles and indigenous knowledge of local residents.

Because of the potential of homestay businesses to provide competitive accommodation, many non-governmental organizations (NGOs) have integrated homestay enterprise development programs into their community-based tourism projects, with the objective of poverty alleviation and sustainable development (Ahmad et al., 2014; Ngo and Doan, 2014); therefore, homestays are community-embedded tourism and hospitality enterprises, as well. Homestay businesses can generate various socio-economic benefits (Pasanchay and Schott, 2021). Homestays businesses are different from multi-national hotel chains, as they provide a source of direct income to homestay owners and local residents from travelers’ expenditure. In addition, the consumption of local products and services during travelers’ stays can enhance the local tourism value chain. In terms of social benefits, homestay business development offers opportunities for the involvement of all family members, cultural exchanges between hosts and guests, and advocacy for social justice (Ahmad et al., 2014). Most importantly, research has found that people of different ages, genders, educational backgrounds, and religious beliefs can become entrepreneurs by establishing homestay businesses (e.g., Ibrahim and Razzaq, 2010; Kunjuraman and Hussin, 2017), making the homestay business model a particularly inclusive development strategy.

The COVID-19 pandemic has severely and adversely affected the global tourism industry (Baum and Hai, 2020). Prompt restrictions on travel have affected the operational processes of a range of tourism and hospitality businesses, including their interactions with customers (Gössling et al., 2021). Likewise, businesses with limited resources and small profit margins, such as are homestay businesses, face more difficulties in recovering (Gössling et al., 2021). It is therefore particularly important to help homestay owners maintain their business and overcome the negative effects of the COVID-19 pandemic (e.g., Cai et al., 2021). However, there is a lack of understanding of how homestay businesses respond and adapt to the effects of – a “new externality” affecting home-sharing businesses in general (e.g., Lim et al., 2021). Addressing this gap creates an opportunity for scholars and practitioners to expand the knowledge needed to assist homestay business owners in times of prolonged crises. Accordingly, this study aimed to explore how homestay businesses transformed their processes during the COVID-19 pandemic.

### Business resilience to external shocks

Research on resilience has grown steadily over the past several decades, representing a coherent body of literature that has evolved and changed with new and emerging understandings. In general, resilience is defined as a system’s ability to maintain and adapt its structure and function in a changing environment (Holling, 1973). Conversely, business resilience is understood based on the identification of organizational resilience and individual resilience, although their boundaries are often blurred (Conz and Magnani, 2020; De Vries and Hamilton, 2021). Organizational resilience is an organization’s ability to generate awareness and reduce vulnerability to risky environments, by reinventing business strategies, adjusting and changing, and responding proactively (Prayag et al., 2020). Resilient organizations are able to survive disruptions, adapt to environmental changes, and transform their processes following external shocks (Pham et al., 2021).

Organizational resilience has been extensively studied in a range of disciplines, including tourism and hospitality contexts (e.g., Hall et al., 2018). In the tourism context, research has mainly focused on different forms of organizational resilience (Jang et al., 2021), but the dominant approach has been to study tourism organizational resilience to external events such as natural disasters (e.g., Prayag et al., 2020). The COVID-19 pandemic has encouraged many researchers to explore the industry’s coping mechanisms from different perspectives (e.g., Pathak and Joshi, 2021; Schwaiger et al., 2022) and in different tourism sectors (e.g., Do et al., 2021).

The literature on individual resilience is mostly from a psychological perspective. The way individuals in different contexts react to disasters and crises has attracted a great deal of scholarly attention (e.g., Hall et al., 2018), and in the business management context, individual resilience and entrepreneurial success has been a popular topic over the past few decades (e.g., Chadwick and Raver, 2020; Fisher et al., 2016). The resilience of entrepreneurs is based on a combination of different personal and behavioral qualities, including their “survival capital” which is an entrepreneur’s ability to sustain a business through unforeseen disruptions; survival capital has been described as “a type of social capital akin to resilience capacity” (Ayala and Manzano, 2014: 128). It has been postulated that organizational resilience is shaped by the ways individual entrepreneurs mobilize their individual survival capital, coping mechanisms, and self-efficacy, during and after disaster/crisis contexts (Ayala and Manzano, 2014; De Vries and Hamilton, 2021; Fang et al., 2020).

### Adaptation strategies during and recovery strategies after crises

Adaptation is a dimension of resilience that refers to the set of actions that individuals or organizations undertake to maintain the capacity to deal with changes, surprises, and system renewal (Daft and Weick, 1984; Ortiz-de-Mandojana and Bansal, 2015). Diverse typologies of adaptation have been proposed in the business management literature, such as those based on purpose and mode of implementation (Hall et al., 2018; Smit and Pilifosova, 2003). In this study, Levinthal’s (1994) definition is adopted: change in a significant organizational attribute that highlights the adaptive cycle/process through which the organization progresses/transforms through different phases of equilibrium.

Businesses’ adaptation strategies relate to the survival of businesses over time, and include, but are not limited to crises. Research in this field has identified various adaptation strategies (e.g., Conz and Magnani, 2020). Schindehutte and Morris (2001) suggested that the entrepreneur, the organizational context and the external environment are three components of adaptation. Pham et al. (2021) proposed a conceptual framework of building business resilience to external shocks, highlighting the role of social networks in building short-term and long-term resilience among small tourism and hospitality businesses. Jiang et al. (2019) focused on the dynamic capabilities of tourism organizations to respond to disruptive environmental changes through a process of routine transformation, resource allocation, and utilization. Dynamic capability is the mechanism that enables businesses’ adaptation to achieve short-term survival during crises (Jiang et al., 2019).

A review of the extant literature found a diverse range of business recovery strategies in post-disaster settings. Many factors influence the strategies adopted, such as the characteristics of the business, external environment, and the operators’ personal beliefs (Dayour et al., 2020). Taking an entrepreneurial marketing perspective, Morrish and Jones (2020) highlighted opportunity-seeking, resource-organizing, creating customer value, and accepting risk, using the post-earthquake experiences of small business entrepreneurs as an example. In the tourism context, post-disaster and post-crisis recovery strategies for tourist destinations have attracted significant attention from scholars (e.g., Mair et al., 2016), and it is believed that crisis preparedness and gaining a better understanding of the consumers’ and entrepreneurs’ responses are vital strategies for recovery.

## Methodology and methods

This study employed a qualitative research methodology (see Creswell, 2007), as little was known about how homestay businesses adapt to the impacts of a pandemic, and how they might recover from the inevitable disruptions caused. Qualitative research focuses on “describing and tracking discourse, including words, meanings, and themes over time” (Altheide et al., 2008: 127). The study was guided by a constructivist paradigm, in which the interaction between researchers and research participants is considered in the process of developing the constructed knowledge (Creswell, 2007). Communication between research partners enhanced the knowing of mind-dependent realities.

Data were collected through in-depth semi-structured interviews with homestay owners located in Vietnam. Purposive sampling was utilized based on the primary researcher’s judgment, with the goal of recruiting information-rich research participants, so their background knowledge “legitimized spending time on the interview” (see Brinkmann and Kvale, 2015: 171). Initially, 40 owners of homestay businesses that were still operational during the pandemic were invited to participate in the study. Of these, 17 accepted the invitation, and provided voluntary and informed consent to participate in online interviews via Skype, Facetime, or Zoom video-conferencing software at the end of 2020. Although the use of online interviews has some limitations, such as the lack of personal contact and potential disruptions due to technical constraints (Salmons, 2014), it was a necessary and convenient approach to collecting data without breaking health and safety protocols, and social distancing requirements. More importantly, it was also essential for the research team to respect the preferences of the interviewees and organize the online interviews according to their interests and comfort with video conferencing applications. Table 1 shows the demographic characteristics of the interview participants and the status of their homestay businesses at the time of the study.

**Table 1.** Interviewees’ demographic profile and business status.

Interviewee	Gender	Years in business	Location	Business size (No. of rooms)	Service offered^a^
#1	F	7	Mekong Delta	7	Room rental only
#2	F	2.5	Mekong Delta	12	Room and boating services
#3	M	11	Mekong Delta	18 (two businesses)	Almost all services except folk arts performance
#4	F	9	Mekong Delta	10	Room rental only
#5	M	16	Central Highland	5	Almost all services except reading room
#6	M	7	Central Highland	15	Fully operational
#7	M	13	Central Highland	14 (two businesses)	Room and motorbike rental
#8	F	6	Central Coast	7	Almost all services except family dinner
#9	F	5	Central Coast	9	Room rental only
#10	F	7	Central Coast	5	Room rental for long-term guests only
#11	M	13	Central Coast	18	Room rental only
#12	F	6	Central Coast	7	Almost all services except massage
#13	M	12	Central Coast	5	Almost all services except reading room
#14	F	9	North Highland	4	Fully operational
#15	M	9	North Highland	7	Almost all services except playing with pets
#16	F	7	North Highland	7	Almost all services except family gathering for traditional drinks
#17	M	12	North Highland	16 (two businesses)	Room rental only

^a^Note: as of December 2020.

A semi-structured interview protocol guided the interviews. The interview protocol provided for a series of open-ended questions focused on the changes resulting from the COVID-19 pandemic, and the adaptive solutions of the homestay owners toward these changes. Semi-structured interviews with open-ended questions gave interviewees the opportunity to express their opinions, while encouraging unanticipated statements and stories to emerge from the conversations (see Marshall and Rossman, 2011). Data saturation was reached, and no additional novel information emerged when interviewing the last few participants.

All interviews were conducted, recorded, and transcribed in Vietnamese – the native language of the primary investigator and interviewees. The primary investigator grew up in Vietnam and has had extensive hospitality industry work experience, so was thus familiar with the local culture and the development of the industry, and had good networks in the research context; this was helpful for interpreting the participants’ responses and “ensur [ing] first-hand knowledge” (Paraskevaidis and Andriotis, 2017: 30) was obtained. Furthermore, this researcher was acutely aware of the cultural challenges facing Vietnamese participating in this research (see Doan et al., 2020). Therefore, the primary investigator employed various communication strategies (e.g., using standardized language, asking and listening sequences, expressing gratitude, etc.) to comfortable conversations with participants (see Doan et al., 2020). Each in-depth interview lasted between 30 and 90 min, and were digitally recorded with the consent of the participants. After transcribing the recordings in Vietnamese, they were translated into English by the primary investigator. During the translation process, the data were cleaned, and repeated and incomplete responses removed.

The interview data were transcribed verbatim, and interpreted and analyzed with the support of NVivo 12 data management software. The primary investigator read the data without taking any notes. After the data had been translated, a systematic coding process was conducted with the co-investigators, to maintain the internal consistency of the findings and analysis. The six stages of thematic analysis of Braun and Clarke (2006) were applied in the data analysis process. Informed by constructivism, an inductive approach to thematic analysis was performed resulting in emergent insights. The participation of three investigators in the data analysis served as the primary validation strategy for this qualitative study (see Creswell, 2007), as this approach enabled corroboration of insights from multiple analysts.

## Findings

To paint a picture of how the homestay businesses adapted during the pandemic, we gathered information about how these businesses operated before the crisis. Apart from delivering accommodation and food services as their core products, the businesses had offered diverse guest experiences, including tours, spa packages, traditional cooking classes, boating, fishing, and farming, prior to the COVID-19 pandemic. However, the pandemic had induced changes in the business environment (e.g., lack of international visitors, hygiene and sanitary restrictions, and other government-imposed restrictions) prompting the operators to adapt their practices. Such adjustments were captured in the themes, which are discussed next.

### Adapting operational protocols

Findings comprising this theme pertained to the immediate responses of the homestay businesses in the face of external shocks. Such strategies are implemented in order for these businesses to continuously operate and survive in the COVID-19 pandemic. In responding to safety requirements imposed to protect people during the pandemic, the homestay owners had to follow instructions from local health authorities. Firstly, close contact such as body touching, shaking hands, and hugs to welcome or farewell people, were minimized or eliminated. Following these hygiene protocols changed the hospitality services of the homestay hosts:

We usually welcome our guests with big smiles and hugs to our home but now we cannot do those anymore [...]Now we stand away from guests. I feel very sorry to welcome guests in that way. It does not show our hospitality. (#9)

Another participant mentioned that close interactions were previously common, not just between their family and guests, but also between their pets and guests.

Guests love our pets. We have two cats and three dogs. We used to let them [pets] play with our guests. [...] but now some guests say that they worry the pets may contract the virus if many people play with them. So we cannot keep our pets here any more. (#15)

This first set of adapted operational protocols can be understood as the homestay owners’ reactive responses (e.g., see Mayson and Barrett, 2017) to the restrictions set out by government institutions during the pandemic. As in many counties, homestays’ compliance with the restrictions imposed by governmental agencies with legitimate authority to handle the pandemic is crucial, so that these businesses can stay operational.

The second important adjustment by the homestay owners, was to stop interactions between other family members and their guests. Guest interactions with the hosts’ family members, except with key staff, became no longer on offer. The owners of homestays #8 and #9 had to move their families out of their homestay vicinities to prevent close contact with guests, and therefore reduce their risk of catching the virus. Social distancing was applied, and the businesses operated with a minimum number of workers.

… only I stay here if we have guests. My wife is pregnant. I moved her and our children to live with my in-laws’ family. We must welcome guests to earn money, but my family is in danger as we do not know if they have had close contact with people at the airport or where they are from. Only me living here is fine, as we do not have many guests. (#8)

Social and physical distancing between guests and host families was widely practiced. Different spaces for guests to enjoy with the families were reduced to ensure there is minimal interpersonal contact.

Our family used to spend time with our guests in the living room and front yard. Now it’s just my wife, my older son, and I working with guests. The rest of the family does not have contact with guests. (#1)

In addition to these accounts, the disruption of personal contact between the host family and guests led to a limitation of guests’ local cultural experiences and inconvenience for the host families. Homestay owner #2 highlighted that a cultural demonstration her mother usually made to guests during their stays could not continue during the pandemic.

Our guests really love my mother. … She taught them how to make betel leaves and areca nuts [a traditional practice for the elderly]. She really enjoys it. With this situation, we do not dare to let her do it any more. (#2)

### Modifying products and service offerings

Since business processes including operational protocols are integrated with the types of product and services of homestay businesses, such value offerings had to be modified. Homestay owners resorted to these modifications mainly to reduce extra services that involve gatherings. These services included cooking classes, sharing meals and facilities, and tours. Homestay owner #9 stated that:

Our cooking class is quite famous here, and our foreign guests really like it. We did it in our kitchen and enjoyed the food together with my family… But at this time, we cannot do it that way as it will be risky for my family and guests. (#9)

Homestay owner #17 rescinded their usual offer of sharing meals with guests while managing to sell their accommodation.

“Meals with family are part of the service in our package. That made us special and people talked about us on TripAdvisor. We cannot serve meals as this will share the same space with my family.” (#17)

There was also a pause in tours organized by the homestay owners, due to guests’ concerns about the risks involved in close contact with others.

We used to organize tours for our guests, but these cannot work now. Guests do not want to share the same van with others. Everyone is worried about having close contact with other guests. (#9)

Withdrawing such services was not a mandatory requirement of the health authorities. Participants explained that these actions were mostly their own proactive solutions for adapting to the pandemic. However, various concerns about losing the uniqueness of homestay services were described, as the authenticity of the experience of guests had diminished.

Homestays offer family-oriented hospitality with individualized experiences for guests. For Homestay owner #16, their occupancy rate was satisfactory as they had been receiving an adequate number of guests. Their main concern was the lack of family ambience, as this made marketing their business harder; thus, posing a new challenge for the business.

The business right now is not fine. If we only sell the room like this, nothing makes us special. Then how can we do marketing and attract guests to come to us? The family ambience at our house is special but we cannot do it. (#16)

With some homestays, where the booking package included welcome massage sessions (e.g., homestay owner #12), canceling this offer had significantly affected their business. While homestay enterprises with limited financial and trained human resources already face many challenges in promoting and maintaining their market (Kunjuraman and Hussin, 2017), the loss of individualized services may subsequently reduce customer satisfaction:

We offered our guests a welcome package. We served them our homemade welcome drink, warm face towel and shoulder massage… Now this package has to change. We cannot do the massage, which our guests like a lot. (#12)

It was clear that homestay owners did not only modify their products and services to meet health restrictions, but also to meet the changing nature of the market, which was now mostly composed of domestic tourists, as a result of the pandemic.

The domestic market is really different from the international market. […] International tourists can spend more time at our homestay, and enjoy our cooking class or tours, but the domestic guests do not spend much time with us during their stay. (#10)

Therefore, efforts to sell extra services to domestic guests were frustrated by their lack of interest. Additionally, domestic guests only stayed for a short term, or shortened their bookings, as the homestays lacked the entertainment facilities of hotels. In response to this problem, Homestay owner #3 tried to offer free bicycle rentals as a solution to entertain guests, but this had not been as successful as expected:

The current business is very lean as guests stay with us [just] for a night, as they have nothing to do with us. I even offer free bicycles, but it does not help much. (#3).

### Reinstating the authenticity of the homestay experience

There was a strong belief among participants that enriching cultural interactions between host families and guests was the most important component of a unique homestay experience in the recovery from the pandemic. To maintain the competitiveness of each homestay, it was highlighted that homestay owners needed to co-create unique interactive experiences with guests from their resources, drawing on (for example) their lifestyle, house design, and entertainment facilities.

Many homestay owners believed that the cultural dimension of the homestay experience differentiated their businesses from other accommodation options. This cultural dimension was strongly perceived to sustain customer satisfaction and address future market preferences. Interviewees explained that the experience with family traditions made guests’ experiences truly authentic.

I do not agree with some places being called a homestay that are just offering accommodation like hotels. A homestay is where guests stay in a house with a family, where they will share their daily activities together. … These can only happen in a house where you live with locals. Hotels cannot offer the same experience. (#5)

In tune with homestay owner #5, homestay owner #11 added that cultural exchanges played a vital role in establishing the emotional connection between guests and the host family.

I try to make my homestay here special. People will not come [just] to stay in the room but for the interaction with the host family. My children love to learn English and interact with international tourists. I can see it is not an opportunity for making money but cultural exchange. And tourists love it. (#11)

Moreover, the very nature of the homestay business was perceived to be their advantage. The owner of homestay #13 believed that they were able to adapt to the crisis promptly due to the small size of homestay businesses, which made it easier to re-organize their unique interactive services for when the pandemic came under control:

The competitive advantage of the homestay business is its small size, not like hotels or resorts. It would be more difficult for them to have something authentic like us. They also cannot change things quickly like us. … I believe any guests come to my place to experience Vietnamese culture with my family. (#13)

To exemplify the solutions for regaining the customers’ cultural experience, homestay owners #1, #2, and #13, encouraged all members of their family to interact with guests as their first recovery strategy. From their daily activities to specialized services for guests, they wanted their guests’ stay to be memorable because of the cultural experience.

We treat our guests like family guests to offer them the experience with us. We can chat, have fun together in the living room or front yard. Guest can hang out there the whole day when my children and my parents join them. My parents show them how to make coconut hats. (#1)

My husband shows them how to do fishing with our round boat. It is a very interesting experience for international tourists. My son takes them to the rice field to fly kites. They love it and recommend new customers to us. (#2)

My mother has lived here for a long time. Her stories of the war are very engaging for our guests. Tourists love to take photos with her. (#13)

These narratives emphasized the significance of reviving social interactions and creating cultural experiences in the homestay servicescape. Ultimately, the delivery of socio-cultural experiences is what differentiates staying in homestays (e.g., Mura, 2015), which was reflected in a proposed strategy relating to the homestay building design. It was believed that adopting the architectural design of a traditional Vietnamese house could enhance the attractiveness of a homestay.

To adapt with the change in market, I think the solution may be in the design of the house. …. As a homestay, the design of the house can be an attractive characteristic to make people feel special staying here. (#16)

Luckily, my parents’ house is a very traditional Vietnamese house, which young people want to take photos of and check in on Facebook. … I try to maintain all the furniture as it used to be. I will get more Vietnamese antiques to decorate our house. (#13)

Recognizing their community-embedded nature, the homestay owners also suggested improving their partnership with their neighbours to organize local cultural and economic activities to entertain their guests. It was believed that the more creative options there were on offer, the better the experience guests could have during their stay with a local host:

My family does not have a farm, but I am working with our neighbors so that our guests can join them in farm work. Dalat is the city of flowers and veggies. The experience on a farm could be cool for tourists spending a day helping to collect roses. I mean everyone needs to be creative and smart to find solutions for their business. (#6)

## Discussion and conclusions

**Figure 1.**
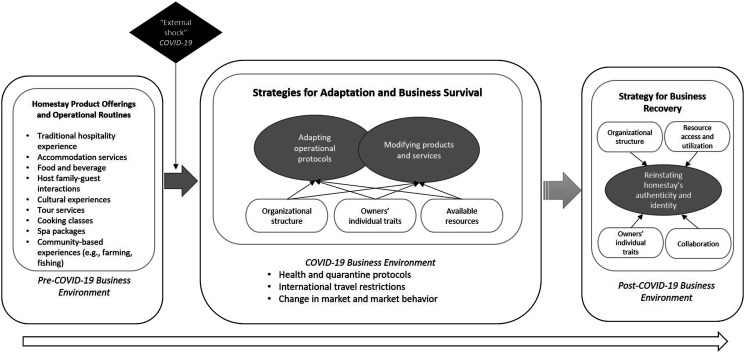
Strategies for adapting to and recovering from the COVID-19 pandemic: Insights of Vietnamese homestay owners.

Homestays are one of the many businesses affected by the COVID-19 pandemic – a prolonged crisis that has disrupted and transformed the tourism and hospitality industry. This study sought to explore homestay businesses’ strategies and the owners’ insights into adapting to and recovering from the COVID-19 pandemic, by investigating changes made to their service delivery practices. Figure 1 illustrates the transformations in Vietnamese homestays’ processes following the emergent changes in the external environmental circumstances created by COVID-19.

Consistent with existing conceptualizations (e.g., Pham et al., 2021) and empirical examinations of small tourism and hospitality businesses in crises (e.g., Dahles and Susilowati, 2015; Fang et al., 2020), the homestay businesses were found to employ and continuously change their products, services, and operational protocols, to stay in business during the pandemic. The first theme that emerged from the analysis, *adapting operational protocols* (e.g., social distancing and limiting service interactions), was consistent with Mayson and Brett’s (2017) discussion of reactive responses to external shocks. In other words, changes in measures enacted by the homestay businesses are indicative of their reactive attributes (e.g., Conz and Magnani, 2020) immediately following the changes in external environmental factors induced by the pandemic.

Furthermore, this resultant theme indicated the homestay businesses’ adaptive attributes and capacities (e.g., Conz and Magnani, 2020; Jiang et al., 2019) during the prolonged crisis, which was shown in the voluntary isolation of family members from guests, and pausing supplementary tourism product offerings. It is suggested that these voluntary actions were also influenced by the homestays’ internal environmental factors (e.g., Jiang et al., 2019; Pham et al., 2021), such as their organizational structure, owners’ cognition and decision-making, and available resources. Homestays are family-based enterprises, and as highlighted in the interviews, the opportunity to interact with hosts’ family members is a key feature of the homestay experience. Given their family-embeddedness, it was critical for homestay owners to balance business continuity and to preserve the health and wellbeing of their families during the pandemic. The absence of social interactions as a homestay resource, affected their operational practices and led to the modification of service offerings. Again, decisions to adjust practices depended on the homestay owners’ awareness and cognition of the changed environmental circumstances (e.g., the risk of getting seriously ill), resonating with the findings of studies that focused on small tourism business owners’ coping mechanisms during (e.g., Schwaiger et al., 2022) and after crises (e.g., Fang et al., 2020).

Since homestay operational practices are strongly linked to their products and experiences (Figure 1), these offerings also had to be adapted. As captured in the second theme, *modifying products and services*, the majority of homestays offered only rooms for rent, and some had limited or ceased offering supplementary tourism and hospitality experiences. These adaptation strategies were not only caused by government-imposed restrictions, but also by changes in the market, to mainly domestic tourists. As highlighted in the findings, domestic tourists’ stays were shorter, and their patronage of supplementary tourism products (e.g., cooking classes, massages, tours, and traditional drinking rituals) was less than that of international tourists; this was likely because the Vietnamese homestay products (e.g., cultural experiences) were somewhat less novel for domestic tourists. Nonetheless, this change in external circumstances inevitably led homestay owners to market switching, which was evident in how they modified and introduced new products and service offerings, a common practice amongst tourism and hospitality MSMEs adapting to crises (e.g., Dahles and Susilowati, 2015; Santiago et al., 2021).

It can also be analyzed that the modification and development of supplementary tourism services also reflected the homestay businesses’ adaptive attributes (e.g., Conz and Magnani, 2020). However, the homestay owners in this study were in an ongoing process of selecting the best product iterations to suit and create the value for the domestic market. In line with Jiang et al.’s (2019) proposition, the homestay businesses were developing new operational routines, albeit in a trial-and-error manner (e.g., the failed initiative to offer free bicycle rental in the *modifying products and service offerings* theme), due to ongoing restrictions, limited resources, and ongoing uncertainties. Nevertheless, it can be explicated that the deployment of these ideas was shaped by the homestay owners’ understandings of the current problem, and supported by their entrepreneurial inclinations (e.g., Fang et al., 2020; Morrish and Jones, 2020).

While this study recognized that the post-pandemic business environment is uncertain, the homestay owners envisioned strategies they could implement to recover from the crisis, as encapsulated in the third theme, *reinstating homestay’s authenticity and identity*. Host and guest interactions in this study were limited to the commercial dimension of hospitality, which had negative consequences on the competitive advantages of the homestay experience (e.g., family-oriented hospitality, cultural experiences, and personalized services). As a result, these limitations diminished the projected authenticity and character of the homestay businesses. Transitioning into a post-pandemic tourism industry (see Figure 1), the homestay owners suggested going back to the original homestay concept, that is, a culturally authentic and immersive experience. On one hand, reinstating the original homestays’ concept as a recovery strategy is not consistent with the idea of “system renewal” fostered in the concepts of business resilience and adaptation (e.g., Daft and Weick, 1984; Ortiz-de-Mandojana and Bansal, 2015). However, this recovery strategy is a plausible option, since culture-seeking international tourists are anticipated to visit Vietnam again after the pandemic. Nevertheless, the success of this business recovery strategy would rely on the homestay owners’ entrepreneurial traits, including their creativity and capacity to innovate (e.g., Fang et al., 2020; Morrish and Jones, 2020).

Consistent with resource-based paradigms for building the resilience of tourism and hospitality MSMEs post-crises (e.g., Pham et al., 2021), Vietnamese homestay owners explained that implementing this recovery strategy would be reliant on access to and utilization of several resources: physical (e.g., for redesigning homestay facilities); cultural (e.g., for re-incorporating cultural dimensions into the homestay experience); financial (e.g., monetary resources to implement ideas); and social (e.g., collaboration within the family and with other homestay owners). For example, the findings emphasized the need to capitalize on the owners’ bonding and bridging social networks, which are essential resources for developing organizational resilience, as noted in prior research on small tourism businesses (e.g., Fang et al., 2020; Pham et al., 2021). The findings showed that because of their structure as family-embedded organizations, the participation of family members in homestay business activities, and the importance of strengthening bonding social capital within each family as a unit was vital. Furthermore, homestay businesses can also be understood as business organizations that are embedded in, and competing with, similar businesses within a community. The participants viewed that collaboration among neighbors was imperative for the delivery of authentic tourist experiences in a community (as a destination) post-pandemic, emphasizing the importance of strengthening their bridging social capital. However, the importance of collaboration initiatives to adaptation strategies during the pandemic was not evident in the data, a finding that diverges from recent studies of family tourism and hospitality MSMEs (e.g., Santiago et al., 2021).

Overall, the Vietnamese homestay businesses showed adaptive capacities to disturbances and changes in environmental circumstances induced by the COVID-19 pandemic, evidenced in the ways they had transformed their processes and practices. Continuous efforts to enhance the homestay experience and create value for the predominantly domestic tourism market were also evident. Yet there was a strong desire among participants to reclaim the authenticity of the homestay experience by resuming business-as-usual service delivery practices and incorporating further cultural elements in their products as soon as possible. Apart from the changing external environmental, several factors (e.g., organizational structure, homestay owners’ individual traits, availability of/access to resources, and collaboration) were proposed to shape homestay businesses’ successful implementation of strategies for adapting to and recovering from the pandemic.

### Implications

This study provides the first attempt to understand homestay businesses’ strategies for adaptation to the effects of the COVID-19 pandemic. By probing into the perspectives of Vietnamese homestay owners, the study contributes to knowledge of tourism and hospitality MSMEs’ resilience to external shocks. Moreover, the findings provide empirical evidence supporting existing conceptualizations of small tourism business resilience building, during and after crises. In practical terms, the study also reveals insights into the adjustments needed to service delivery, and the practices implemented by homestay business owners responding to health-related crises such as COVID-19. The findings suggest that timely changes to operational routines and, most importantly, family arrangements (e.g., daily set-up), are crucial for homestay and other home-sharing business operators in the early stage of a crisis.

The findings of this study also reveal insights into the essential resources needed by homestay businesses to recover from the impacts of a pandemic. Since homestay businesses in Vietnam and other parts of Southeast Asia are embedded in livelihood development initiatives (e.g., Dahles and Susilowati, 2015), these findings have implications for stakeholders working towards homestay businesses’ recovery from the COVID-19 pandemic. Specifically, the findings direct proponents of homestay business and community-based tourism development programs to assist homestay operators in securing the resources (e.g., funding) needed for reviving these businesses. These stakeholders can also deliver programs to enhance collaboration amongst homestay owners within their host communities.

### Limitations

Since this study was qualitative, the findings cannot be generalized, and are limited to the perspectives of Vietnamese homestay business owners. The data were cross-sectional and collected during the first year of the COVID-19 pandemic, which means the impacts of the subsequent variants of the virus were not taken into account. Researchers should conduct longitudinal inquiries focusing on how homestay businesses adapted during later surges of the COVID-19 pandemic. Future research can also empirically examine relationships between resource use and business resilience constructs using quantitative approaches. Since homestay businesses are embedded within families and communities, researchers can also adopt concepts of family entrepreneurship and sustainable livelihoods frameworks in future investigations.
